# Isolation method and xeno-free culture conditions influence multipotent differentiation capacity of human Wharton’s jelly-derived mesenchymal stem cells

**DOI:** 10.1186/scrt232

**Published:** 2013-07-11

**Authors:** Maria Cristina Corotchi, Mirel Adrian Popa, Anca Remes, Livia Elena Sima, Ilinca Gussi, Marilena Lupu Plesu

**Affiliations:** 1 Angiogenesis and Cardiovascular Remodeling Group, Department of Regenerative Medicine, Institute of Cellular Biology and Pathology “Nicolae Simionescu” of the Romanian Academy, 8 B.P. Hasdeu Str., 050568, Bucharest, Romania; 2 Department of Molecular Cell Biology, Institute of Biochemistry of the Romanian Academy, 296 Splaiul Independentei, 060031, Bucharest, Romania; 3 Department of Obstetrics and Gynecology, Clinical Hospital “Dr. Ioan Cantacuzino”, 5-7 Ion Movila Str., 020000, Bucharest, Romania

**Keywords:** Wharton’s jelly, Mesenchymal stem cells, Xeno-free medium, Plasticity, Endothelial differentiation

## Abstract

**Introduction:**

Human Wharton’s jelly (WJ) has become a preferred source of mesenchymal stem cells (MSCs) whose clinical applications are limited by the use of adequate xeno-free (XF), *in vitro* manipulation conditions. Therefore, the objective of our study was to characterize WJ-derived MSCs (WJ-MSCs), isolated by different methods and cultured in a commercially available, MSC XF medium, not least of all by investigating their endothelial differentiation capacity.

**Methods:**

WJ explants and enzymatically dissociated WJ cells were cultured in a defined, XF medium for MSCs. Adherent cells at passages 2 and 5 were characterized as MSCs by flow cytometry, MTT, real-time quantitative reverse transcription PCR, and functional multipotent differentiation assays. The endothelial differentiation capacity of MSCs isolated and expanded until passage 2 in the MSC XF medium, and then subcultured for five passages in a commercially available endothelial growth medium (group A), was assessed over serial passages, as compared to adherent WJ-derived cells isolated and expanded for five consecutive passages in the endothelial medium (group B).

**Results:**

The MSC phenotype of WJ explant- and pellet-derived cells, isolated and expanded in the MSC XF medium, was proven based on the expression of CD44/CD73/CD90/CD105 surface markers and osteo-/adipo-/chondrogenic multipotent differentiation potential, which differed according to the isolation method and/or passage number. Upon exposure to endothelial differentiation cues, cells belonging to group A did not exhibit endothelial cell characteristics over serial passages; by contrast, WJ pellet-derived cells belonging to group B expressed endothelial characteristics at gene, protein and functional levels, potentially due to culture conditions favoring the isolation of other stem/progenitor cell types than MSCs, able to give rise to an endothelial progeny.

**Conclusions:**

The use of defined, MSC XF media for isolation and expansion of human WJ-MSCs is a prerequisite for the establishment of their real endothelial differentiation capacity, as candidates for clinical therapy applications. Thus, the standardization of WJ-MSCs isolation and culture expansion techniques in defined, MSC XF media, for their accurate characterization, would be a priority in the stem cell research field.

## Introduction

Among multiple sources of stem cells, human umbilical cord matrix, namely, Wharton’s jelly (WJ), has recently become the preferential source of stem cells, including mesenchymal stem cells (MSCs), because of rapid availability with a large donor pool, non-invasive collection with no risk or discomfort for the donor, no ethical constraints, high *in vitro* expandable rates and multipotent differentiation potential [1-7]. Due to proven immunomodulatory effects, WJ-derived MSCs (WJ-MSCs) are now considered attractive agents not only for autologous, but also for allogeneic cell therapy approaches of malignant and non-malignant, hematopoietic and non-hematopoietic, inherited and acquired diseases [1,8,9].

Whereas adult bone marrow (BM)-derived MSCs (BM-MSCs) have shown limited therapeutic benefits for organ regeneration, it has been postulated that WJ-derived primitive stromal cells are a valuable alternative source of cells that possess multipotent properties between embryonic and adult stem cells [2,10-12]. WJ-MSCs have a higher proliferation rate [13,14] and a higher expression level of early endodermal markers, as well as undifferentiated human embryonic and pluripotent/stem cell markers, both at early and late passages [12]. Although WJ-MSCs share common surface markers with BM-MSCs, such as the immunomodulatory molecules [4,15], they are endowed with superior plasticity properties [3]. Furthermore, it has been shown that the immune privilege exerted by WJ-MSCs is also maintained in the differentiated adipogenic, osteogenic and chondrogenic progeny [5].

Generation of an endothelial cell outgrowth from the matrix of the umbilical cord, for vascular regeneration purposes, has been described by several groups [13,16-19]; however, the applied differentiation protocols did not involve the use of a defined MSC medium for WJ-MSCs isolation prior to their seeding into endothelial differentiation media, raising the question of potential contamination of the generated cultures with other stem cell types able to give rise to an endothelial progeny, circulating endothelial progenitor cells or mature endothelial cells.

Several groups have established various protocols for the isolation and characterization of stromal cells from WJ [11,18,20-23]. However, the effects of defined, xeno-free (XF) media, designed for MSCs isolation and expansion, on the gene, protein and functional profiles of WJ-MSCs have not been thoroughly investigated. It has been shown that XF culture systems allow for better multipotent differentiation and/or expansion rates of adipose tissue- and BM-MSCs, serving as a preferred alternative to fetal bovine serum (FBS)-containing media for the production of large scale, functionally competent, clinical grade MSCs [24-26]. In addition, the use of FBS for MSCs *in vitro* isolation and expansion raises concerns for the transmission of zoonoses and induction of immunogenic reactions after clinical transplantation, due to xenogeneic proteins transmitted from FBS to MSCs during culture [27,28]. Therefore, the *in vitro* manipulation of MSCs by using XF culture conditions before clinical applications has become an important step in order to yield homogenous cell populations with self-renewal and multi-lineage differentiation potential, but without an increase in chromosome aberrations.

Despite a wide range of potential clinical applications, such as for bone [29], cartilage [30], musculoskeletal [31,32] and nerve [33] regeneration, for the treatment of liver fibrosis [10,34] and type 1 diabetes [35], as well as for heart valve [36] and vocal fold reconstruction [37], WJ-MSC populations isolated in defined, XF conditions have not been fully characterized. Therefore, given their particular plasticity and developmental flexibility, the impact of XF conditions on functional properties of umbilical cord stromal cells deserve to be thoroughly examined. To elucidate the real value of WJ-MSCs for clinical cell replacement therapy, further work is thus needed in order to determine whether these cells, isolated and expanded in defined, XF media, truly express differentiation capabilities beyond the canonical plasticity towards the adipogenic, osteogenic and chondrogenic cell lineages [38].

To get further insights into the biology of WJ-derived cells, the objectives of our study were to isolate, by using different protocols, and expand human WJ-MSCs in a commercially available, XF medium, selective for MSCs, and to characterize them at gene, protein and functional levels, not at the least by determining their endothelial differentiation capacity.

## Methods

### MSCs isolation

Human umbilical cord samples were collected following term deliveries at the Department of Obstetrics and Gynecology, “Dr. I. Cantacuzino” Hospital, Bucharest, Romania. The samples were obtained upon written informed consent from mothers and complied with European Union and national legislation regarding human samples collection, manipulation and personal data protection. All samples were tested for the absence of HIV, HBV and HCV and processed within three hours of collection.

**Figure 1:**
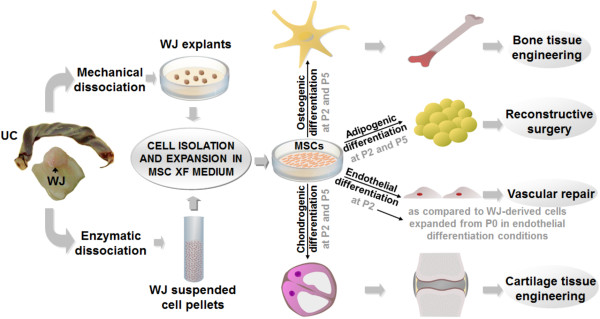
**Experimental design.** Isolation and expansion of MSCs, derived from WJ explants and enzymatically dissociated WJ, in a XF medium defined for MSCs, followed by their characterization, not the least by investigating their osteogenic, chondrogenic, adipogenic (at P2 and P5) and endothelial differentiation capacity (at P2, as compared to WJ-derived cells expanded from P0 in endothelial differentiation conditions), for use in different clinical settings of regenerative and reconstructive medicine [13,16-19,29,30,39]. MSCs, mesenchymal stem cells; P, passage; UC, umbilical cord; WJ, Wharton’s jelly; XF, xeno-free.

WJ was isolated from the umbilical cords after dissection and removal of the umbilical cord arteries, vein and amniotic epithelium. MSCs isolation from WJ was achieved either by mechanical dissociation for generation of tissue explants of approximately 2 mm^2^, or by enzymatic dissociation with collagenase I and hyaluronidase (Sigma-Aldrich, St. Louis, MO, USA) for generation of WJ cell pellets. Tissue explants and suspended cell pellets were thereafter seeded onto tissue cultured dishes (10 tissue explants/dish) and flasks (5 × 10^5^ cells/flask), respectively, pre-coated with MesenCult™-XF Attachment Substrate, in MesenCult™-XF Complete medium (XF medium), consisting of MesenCult™-XF Basal medium supplemented with 20% MesenCult™-XF Proliferation Supplement (all from STEMCELL Technologies™ Inc., Vancouver, BC, Canada), L-Glutamine to a final concentration of 2 mM, 100 U/ml penicillin, 100 μg/ml streptomycin and 50 μg/ml neomycin (all from Sigma-Aldrich). Cultures of both WJ tissue explants and suspended cell pellets were maintained at 37°C with 5% CO_2_ and 21% O_2_ in a humidified atmosphere. For optimal cell growth, medium was changed at the moment of subculture or, if the medium appeared acidic prior to cells reaching 80% confluence, half-medium change was performed. For the purpose of subculturing, the cells were enzymatically-detached using the MesenCult™ Dissociation Kit (STEMCELL Technologies™ Inc.). Cells from passages (P) 2 and 5 were used for cell characterization experiments (see Figure 1 describing the experimental design). Five WJ explant-derived and eight WJ pellet-derived MSC populations were isolated and expanded in the XF medium until P5 for the proposed experiments. All procedures were approved by the Ethical Committee Board of the Institute of Cellular Biology and Pathology “Nicolae Simionescu”, Bucharest.

### Cells characterization

#### Proliferation assays

Morphological characterization of cells was done by light microscopy, using an inverted epifluorescence microscope (Eclipse TE300, Nikon, Tokyo, Japan) and a digital camera system for imaging (Digital Net Camera DN100, Nikon). Viability and proliferation of WJ explant- and pellet-derived MSC populations (n = 5) at P2 were evaluated using a modified colorimetric 3-(4,5-dimethylthiazol-2-yl)-2,5-diphenyltetrazolium bromide (MTT) assay method (CellTiter96 Non-Radioactive Cell Proliferation Assay, Promega, Madison, WI, USA), according to the manufacturer’s instructions. Cells were seeded in 96-well plates at a density of 3 × 10^3^ cells/well in the XF medium. The assay was performed every 24 hours during a two-week period, in triplicates. The absorbance was measured at a wavelength of 570 nm by using a spectrophotometric plate reader (Mithras LB 940, Berthold Technology, Bad Wildbad, Germany).

#### Adipogenesis, osteogenesis and chondrogenesis assays

*In vitro* differentiation potential of WJ explant- and pellet-derived MSC populations (n = 5) at P2 and P5 into adipogenic, osteogenic and chondrogenic lineages was assessed by using specifically formulated cell culture media (MesenCult™ Adipogenic Stimulatory Supplements-Human and MesenCult™ Osteogenic Stimulatory Kit-Human from STEMCELL Technologies™ Inc., as well as StemMACS ChondroDiff Media-Human from Miltenyi Biotec GmbH, Bergisch Gladbach, Germany), and the manufacturer’s guidelines.

Briefly, for adipocyte and osteocyte differentiation, MSCs were plated at a density of 12.5 × 10^3^ cells/cm^2^ and 5 × 10^3^ cells/cm^2^, respectively, in six-well cell culture plates and kept in normoxic conditions for two and four weeks, respectively, with medium changed every three days. Adipogenic differentiation was verified by staining with Oil Red O (0.5% in isopropanol) and osteogenic differentiation was visualized by Alizarin Red S (2% in distilled water) staining (both from Sigma-Aldrich). To these purposes, cells were fixed with a 4% paraformaldehyde solution in 1x phosphate-buffered saline (PBS) for 30 minutes at room temperature; for Oil Red staining only, cells were washed with distilled water and 60% isopropanol. Cells were thereafter maintained with the corresponding staining for five minutes, rinsed with distilled water three times to remove excess stain, and then photographed by using an inverted microscope (Eclipse TE300, Nikon) and a digital camera system for imaging (Digital Net Camera DN100, Nikon).

For chondrogenic differentiation, 2.5 × 10^5^ cells were centrifuged (150 × g, five minutes) at room temperature. The resulting cell pellets were cultured for 24 days in 15 ml polystyrene centrifugation tubes in differentiation medium, with medium changed every 3 days. The resulting nodular cell clusters were embedded in paraffin (Carl Roth GmbH, Karlsruhe, Germany) and 10-μm-thick sections were stained with Alcian Blue (1% in 30% acetic acid) and Toluidine Blue O (both from Sigma-Aldrich) for 30 and 2 minutes, respectively, for chondrogenic extra-cellular matrix containing hyaluronic acids. Briefly, the stained preparations were washed in running water for two minutes, rinsed in distilled water, dehydrated in graded ethanol series, followed by three clarification steps in xylene and mounting. The cover-slipped slides were then photographed by using an inverted microscope (Eclipse TE300, Nikon) and a digital camera system for imaging (Digital Net Camera DN100, Nikon). The negative controls were represented by corresponding MSCs at P5 not cultured into differentiation media.

#### Endothelial differentiation assays

For the purpose of testing the true endothelial differentiation potential of WJ explant- and pellet-derived MSCs, isolated in a highly selective medium for MSCs, we conducted parallel endothelial differentiation assays, by using two cell groups (A and B). Cells from group A (n = 5) were either explant- or pellet-derived MSCs isolated and expanded in the XF medium until P2, and thereafter exposed to endothelial differentiation conditions for five consecutive passages. Cells from group B (n = 5) consisted of adherent cells resulted from either WJ-derived explants or pellets, directly cultured from P0 to P5 in endothelial differentiation conditions. The protocols applied for WJ isolation and mechanical/enzymatic dissociation, as well as for endothelial differentiation, were identical for both cell groups.

Endothelial differentiation conditions consisted of explants/cells seeding onto culture flasks pre-coated with 2 μg/cm^2^ fibronectin (BD Biosciences, San Jose, CA, USA), in Endothelial Cell Growth Medium MV2 (Promocell, Heidelberg, Germany), supplemented with 20% FBS and 40 ng/ml vascular endothelial growth factor (VEGF). The cells were kept in normoxic conditions in a humidified atmosphere for five serial passages. Endothelial differentiation was assessed by morphology, flow cytometry, qualitative reverse transcription (RT) polymerase chain reaction (PCR) and real-time quantitative RT-PCR (qRT-PCR), as well as Matrigel tube formation assay.

#### Flow cytometry analysis

Expression of cell surface molecules on WJ explant- and pellet-derived MSCs at P2 and P5 (n = 5) cultured in the XF medium was assessed by using a FACSCalibur instrument (BD, Franklin Lakes, NJ, USA). Surface marker expression on WJ explant- and pellet-derived cells cultured in endothelial differentiation conditions for five passages (groups A and B, n = 5 for each group) was analyzed by using a MoFlo FACS instrument (Dako, Glostrup, Denmark). The controls were represented by enzymatically dissociated, freshly isolated WJ cells (n = 3) and a NUFF1 human fibroblast cell line. To this purpose, 1 × 10^5^ cells were stained with fluorochrome-conjugated (either phycoerythrin (PE) or fluorescein isothiocyanate (FITC)) antibodies against CD31, CD34, CD44, CD45, CD73, CD90, CD105, CD144 and CD309 (VEGF receptor (VEGFR) 2) antibodies (MACS, Miltenyi Biotec). Enzymatically-detached cells, using the MesenCult™ Dissociation Kit (STEMCELL Technologies™ Inc.) for MSCs and accutase (Sigma-Aldrich) for cells cultured in the endothelial differentiation medium, were washed in 1× PBS containing 2% FBS and incubated for 30 minutes at 4°C with the appropriate diluted FITC-/PE-conjugated antibodies (1:100 dilution for CD90 antibody and 1:50 dilution for the rest of antibodies, in 1× PBS containing 2% FBS). For isotype control, the cells were stained with 1:50 diluted isotype-matched IgG (IgG1, MACS, MiltenyiBiotec). Ten thousand cells were acquired from each sample and flow cytometry data were analyzed using either the CellQuest Pro (BD) or Summit 4.0 (Dako) softwares, according to the instrument used. Median fluorescence intensity (MFI) was measured for each surface marker and background values were subtracted to obtain ΔMFI.

#### RT-PCR and qRT-PCR

Molecular characterization of cells used in differentiation assays was done by RT-PCR and qRT-PCR. For qRT-PCR, total RNA was extracted with the PureLink™ RNA Mini Kit (Life Technologies™, Carlsbad, CA, USA). First-strand cDNA synthesis was performed on 1 μg of total RNA; cDNA samples were thereafter amplified in triplicates by using a real-time PCR system (Applied Biosystems 7900HT Fast, Life Technologies™) for 40 cycles (95°C for 2 minutes, 95°C for 5 sec, 60°C for 10 sec, 72°C for 15 sec, plus a dissociation cycle at 95°C for 15 sec and 60°C for 15 sec) with specific oligonucleotide primers (Applied Biosystems-Life Technologies™, Carlsbad, CA, USA) to assess quantitative mRNA expression for *osteocalcin*, *aggrecan*, *CD31*, *von Willebrand factor (vWF)* and *glyceraldehyde 3-phosphate dehydrogenase (GAPDH)*, as endogenous control. WJ-MSCs not used into differentiation assays and enzymatically dissociated, freshly isolated WJ cells were used as controls; qRT-PCR data were analyzed by using the SDS 2.4 Standalone software (Applied Biosystems-Life Technologies™). For RT-PCR, total RNA was extracted with the GenElute™ Mammalian Total RNA Miniprep Kit (Sigma-Aldrich). First-strand cDNA synthesis was performed on 1 μg of total RNA; cDNA samples were thereafter amplified in a thermocycler (Bio-Rad Laboratories, Hercules, CA, USA) for 35 cycles (94°C for 45 sec, 60°C for 45 sec and 72°C for 45 sec) with specific oligonucleotide primers (Metabion, Martinsried, Germany) to assess mRNA expression for *CD31*, *CD34*, *CD144*, *VEGFR1*, *VEGFR2*, *vWF*, *GATA2*, *Tie-2* and *GAPDH*, as endogenous control. Enzymatically dissociated, freshly isolated WJ cells and a human umbilical vein endothelial cell (HUVEC) line (Promocell) were used as controls. The oligonucleotide primer sequences for both RT-PCR and qRT-PCR are shown in Table 1.

**Table 1:** Sequences of the oligonucleotide primers used for qRT-PCR and RT-PCR

**Gene**	**GeneBank® accession number**	**Sequences of oligonucleotide primers**	**Predicted size (bp)**
**qRT-PCR**			
*vVF*	[NM_000552.3]	S: 5′-CGACATGGAGGATGCCGT-3′	236
		A: 5′-ACTCATTGATGAGGCAGGGGT-3′	
*CD31*	[NM_000442.4]	S: 5′-CTGCTGACCCTTCTGCTCTG-3′	203
		A: 5′-TAAAACAGCACGTCATCCTTATAGA-3′	
*GAPDH*	[NM_002046]	S: 5′-TTGGTATCGTGGAAGGACTCA-3′	270
		A: 5′-TGTCATCATATTTGGCAGGTTT-3′	
*Aggrecan*	[NM_001135.3]	S: 5′-GTGCCTATCAGGACAAGGTCT-3′	167
		A: 5′-GATGCCTTTCACCACGACTTC-3	
*Osteocalcin*	[NM_199173.4]	S: 5′-GCCCTCACACTCCTCGCCCTA-3′	100
		A: 5′-AGGCTGCACCTTTGCTGGACTC-3	
*GAPDH*	[NM_001256799.1]	S: 5′-TTGGTATCGTGGAAGGACTCA-3′	267
		A: 5′-TGTCATATTTGGCAGGTTT-3	
**RT-PCR**			
*CD31*	[NM_000442]	S: 5′-AGCACCACCTCTCACGTCA-3′	250
		A: 5′-CTTGGATGGCCTCTTTCTTG-3′	
*CD144*	[NM_001795]	S: 5′-CCTTGGGATAGCAAACTCCA-3′	283
		A: 5′-CTTTGCCTCCAGGCAGATAG-3′	
*CD34*	[NM_001773]	S: 5′-TCAGTTCTAGTCTCTCTGGGGC-3′	328
		A: 5′-ATAAGGGTTAGGAGCTGATCTGG-3′	
*VEGFR1*	[NM_002019]	S: 5′-CAGCCCATAAATGGTCTTTGCC-3′	557
		A: 5′-TAATTTGACTGGGCGTGGTGTG-3′	
*VEGFR2*	[NM_002253]	S: 5′-GTGACCAACATGGAGTCGTG-3′	660
		A: 5′-CCAGAGATTCCATGCCACTT-3′	
*VWF*	[NM_000552]	S: 5′-CGACTTCCTTACCCCCTCTG-3′	247
		A: 5′-GCAGGAGCACACGTCGTAG-3′	
*GATA2*	[NM_032638]	S: 5′-CCCTAAGCAGCGCAGCAAGAC-3′	439
		A: 5′-TGACTTCTCCTGCATGCACT-3′	
*Tie-2*	[NM_000459]	S: 5′-CATACTGGGGAAAGCAATGAAAC-3′	281
		A: 5′-ACCACTGTTTTTCACCTTCCAAA-3′	
*GAPDH*	[NM_002046]	S: 5′-ACCACAGTCCATGCCATCAC-3′	450
		A: 5′-TCCACCACCCTGTTGCTGTA-3′	

A, antisense; S, sense.

#### Matrigel assay

Matrigel assay was performed by using 50 μl of Matrigel Basement Membrane Matrix (BD Biosciences) added to 48-well plates; the Matrigel was allowed to solidify at 37°C for 30 minutes and 5 × 10^4^ cells were, thereafter, suspended in 100 μl Endothelial Cell Growth Medium MV2 (Promocell), supplemented with 20% FBS and 40 ng/ml VEGF, and plated onto the Matrigel layer. A HUVEC line (Promocell) was used as a positive control. After 24 hours, the medium was removed and the formation of vascular tube-like structures was assessed with an inverted microscope (Eclipse TS100, Nikon) and a digital camera system for imaging (Digital SLR Camera D300, Nikon).

### Statistical analyses

Statistical analyses were performed either by a one-way analysis of variance (ANOVA) with the Microcal OriginPro - version 6.0 software (OriginLab Corporation, Northampton, MA, USA) or by a two-tailed unpaired *t* test with the GraphPad Prism 4 software (GraphPad Software, Inc., La Jolla, CA, USA). The results were presented as mean ± standard error of the mean and differences were considered statistically significant when *P*-value < 0.05.

## Results

### WJ mesenchymal-like cells highly proliferate in the XF medium

**Figure 2:**
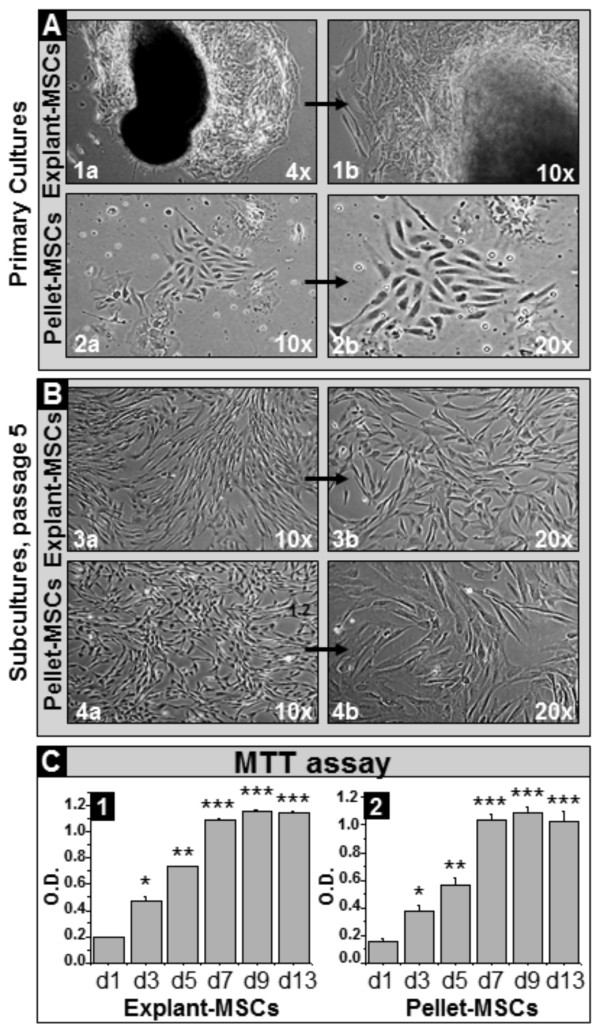
**Assessment of morphology and proliferation rate of MSCs isolated and subcultured in XF medium. A.** Morphological evaluation of primary MSC cultures derived from WJ explants (explant-MSCs -**1a**, **1b**) and enzymatically dissociated WJ (pellet-MSCs -**2a**, **2b**); **B.** Morphological evaluation of P5 subcultures of WJ explant **(3a**, **3b)**- and pellet **(4a**, **4b)**-derived MSCs; higher magnifications of corresponding pictures are pointed by arrows; **C.** MTT assay on WJ explant (1)- and pellet (2)-derived MSCs at P2; d, day of assessment; *, **, ***, *P* < 0.05, indicates statistical significance in cell proliferation during consecutive time points of assessment (*, Day 3 vs. Day 1; **, Day 5 vs. Day 3; *** days 7, 9, 13 vs. Day 5). MSCs, mesenchymal stem cells; P, passage; WJ, Wharton’s jelly; XF, xeno-free.

In order to test whether the XF medium is suitable for WJ-MSCs isolation and expansion, we isolated WJ cells using two different approaches, either from WJ explants or enzymatically dissociated WJ cells. Our results demonstrated that primary cultures of adherent cells, with a mesenchymal-like morphology, grew in colonies out of the WJ explants (Figure 2.A.1a, 1b) and were subcultured, on average, at 14 days after plating, whereas adherent cells resulting from culture of suspended WJ-derived pellets (Figure 2.A.2a, 2b) reached 80% confluence and were subcultured, on average, at 10 days after plating. Both explant and pellet-derived cells maintained their morphology after subsequent subculturing (Figure 2.B.3a, 3b and 4a, 4b, respectively). Furthermore, both cell population types presented a high proliferation rate, which doubled every 24 hours (*P* < 0.05) during the first week of assessment (Figure 2.C). Thus, the XF medium was suitable for the isolation of both WJ explant- and pellet-derived adherent cells with a mesenchymal-like phenotype and a high proliferation rate.

### WJ-derived cells cultured in the XF medium presented MSC characteristics

**Figure 3:**
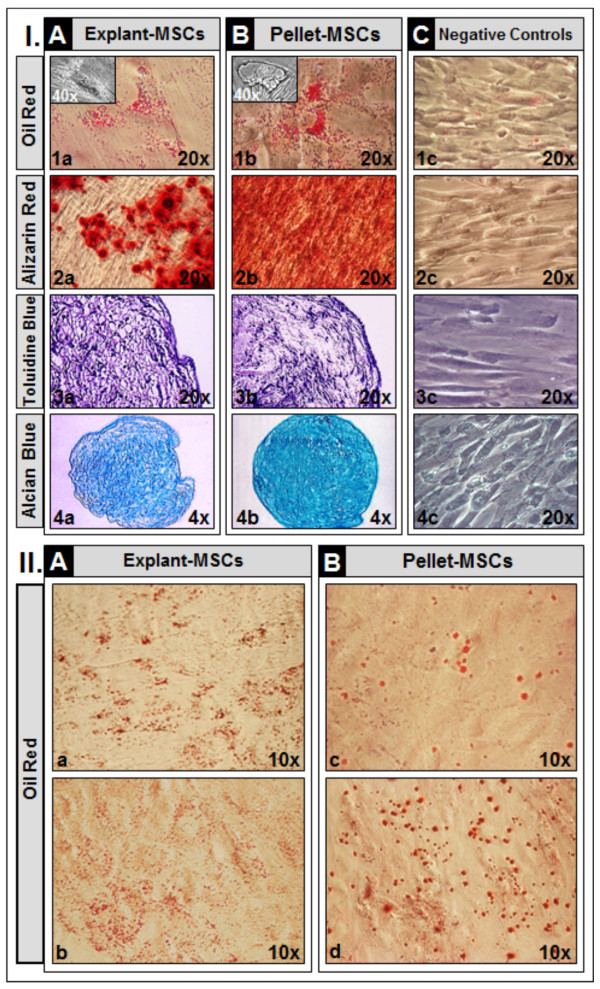
***In vitro *differentiation assays of WJ-MSCs at P2/P5, isolated and expanded in XF medium. I**. Representative images of MSCs, isolated from both WJ explants (explant-MSCs, **A**) and enzymatically dissociated WJ (pellet-MSCs, **B**), showing differentiation towards the adipocyte (A.1a - P5, B.1b - P2), osteocyte (A.2a - P5, B.2b - P2) and chondrocyte (A.3a/4a - P5, B.3b/4b - P2) lineages, as assessed by Oil Red, Alizarin Red and Toluidine Blue/Alcian Blue staining, respectively; **C.** Negative controls, represented by corresponding pellet-derived MSCs not used in differentiation assays and stained with Oil Red (C.1c), Alizarin Red (C.2c), Toluidine blue (C.3c) and Alcian Blue (C.4c); **II.A** and **B**. Representative, lower magnification (10x) images of adipogenic differentiation of WJ explant (a - P2; b - P5)- and pellet (c - P2; d - P5)-derived MSCs. MSCs, mesenchymal stem cells; P, passage; WJ, Wharton’s jelly; XF, xeno-free.

For the purpose of testing if the XF medium could lead to the isolation and expansion of cells with true MSC characteristics, we assessed their functional ability to differentiate into adipogenic, osteogenic and chondrogenic lineages, as well as the presence of MSC molecules at gene and protein levels. The cyto/histochemical staining of *in vitro* differentiation assay preparations revealed that, under specific culture conditions, both WJ explant- and pellet-derived MSCs, isolated and expanded in the XF medium, presented adipogenic, osteogenic and chondrogenic differentiation capacity (Figure 3.I.A, I.B), as compared to controls represented by WJ explant (data not shown) and pellet-derived MSCs (Figure 3.I.C) not cultured in differentiation media that did not show three-lineage differentiation potential. In respect to the degree of adipogenic differentiation, accumulation of lipid droplets was more robust in WJ pellet-derived MSCs at P5, as compared to WJ pellet-derived MSCs at P2 and WJ explant-derived MSCs (Figure 3.II.A, II.B).

Furthermore, quantitative gene expression of *osteocalcin* and *aggrecan*, involved in MSCs differentiation into osteogenic and chondrogenic lineages, respectively, significantly increased (*P* < 0.05) in explant-derived MSCs, as compared to controls and by passage progression. Interestingly, in pellet-derived MSCs, both *aggrecan* and *osteocalcin* gene expression were significantly increased (*P* < 0.05) at P2 as compared to controls, but significantly decreased (*P* < 0.05) by passage progression (Figure 4.1a, 1b).

**Figure 4:**
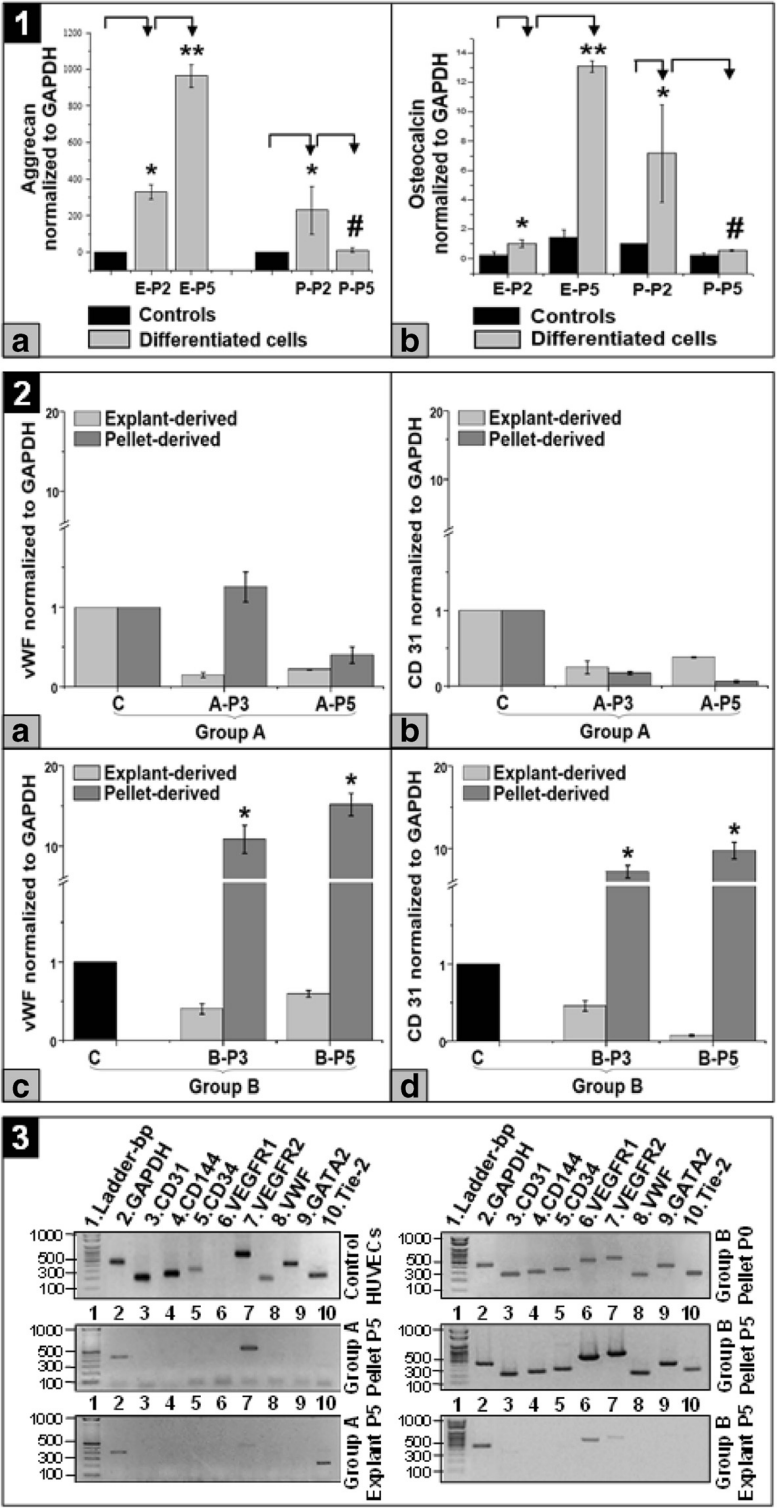
**Gene expression profiles in WJ-derived cells. 1. ***Aggrecan ***(a)** and *osteocalcin ***(b)** quantitative gene expression profiles at P2 and P5 in WJ explant (E-P2, E-P5)- and pellet (P-P2, P-P5)-derived MSCs isolated and expanded in the XF medium, as compared to controls represented by WJ-MSCs at P5 **(a)** and P2/P5 **(b)**, not employed into differentiation assays; * and **, statistically significant increase in gene expression (*P* < 0.05), as compared to control and previous passage, respectively; #, statistically significant decrease in gene expression (*P* < 0.05), as compared to previous passage; **2.** Quantitative *vWF ***(a)** and *CD31 ***(b)** gene expression profiles in explant- and pellet-derived cells belonging to group A at P3 and P5 (A-P3, A-P5, C = Controls represented by explant- and pellet-derived WJ-MSCs not used into endothelial differentiation assay); Quantitative *vWF ***(c)** and *CD31 ***(d)** gene expression profiles in explant- and pellet-derived cells belonging to group B at P3 and P5 (B-P3, B-P5, C = Control represented by enzymatically dissociated, freshly isolated WJ cells, * = statistically significant increase in gene expression (*P* < 0.05) between group B Control (C) and B-P3/B-P5); **3.** Qualitative endothelial gene expression profiles in WJ explant- and pellet-derived cells belonging to groups A and B, after five passages of exposure to endothelial medium, as compared to enzymatically dissociated, freshly isolated WJ cells (pellet-P0) and HUVECs. HUVECs, human umbilical vein endothelial cells; MSCs, mesenchymal stem cells; P, passage; vWF, von Willebrand factor; WJ, Wharton’s jelly; XF, xeno-free.

In addition, flow cytometry analysis of XF medium cultured MSCs, derived from both WJ explants and cell pellets, revealed that the cells were positive, at both P2 (Figure 5.1 and 2, respectively) and P5 (data not shown) for MSC surface markers such as CD44, CD73, CD90 and CD105, and were negative for hematopoietic stem cell (CD34) and monocyte-macrophage (CD45) markers; interestingly, enzymatically dissociated, freshly isolated WJ cells (P0) showed only CD44 surface marker expression (Figure 6.C). When we compared MFI of MSC markers between passages, the WJ explant-derived MSCs had a statistical significantly higher (*P* < 0.05) CD73 expression at P5, as compared to P2 (Figure 5.3a). Furthermore, MSCs derived from WJ cell pellets exhibited a statistical significantly higher (*P* < 0.05) expression of CD44 at P2, as compared to P0 and P5, and a statistical significantly higher (*P* < 0.05) expression of CD73 marker at P2, as compared to P5 (Figure 5.3b). Moreover, when we compared explant- versus pellet-derived MSCs, pellet-derived cells at P2 expressed a statistical significantly higher MFI for CD44 and CD73 markers (*P* < 0.005 and *P* < 0.05, respectively) than the explant-derived counterparts and a statistical significantly higher MFI (*P* < 0.005) for CD44 marker, as opposed to pellet-derived WJ cells at P0 (Figure 5.3c). Taken together, the results of *in vitro* differentiation assays corroborated with the results of MSC molecules assessment indicated that both WJ explant- and pellet-derived adherent cells cultured in the XF medium showed different degrees of multipotent differentiation and expression of markers characteristic for MSCs, according to the isolation method and/or passage number.

**Figure 5:**
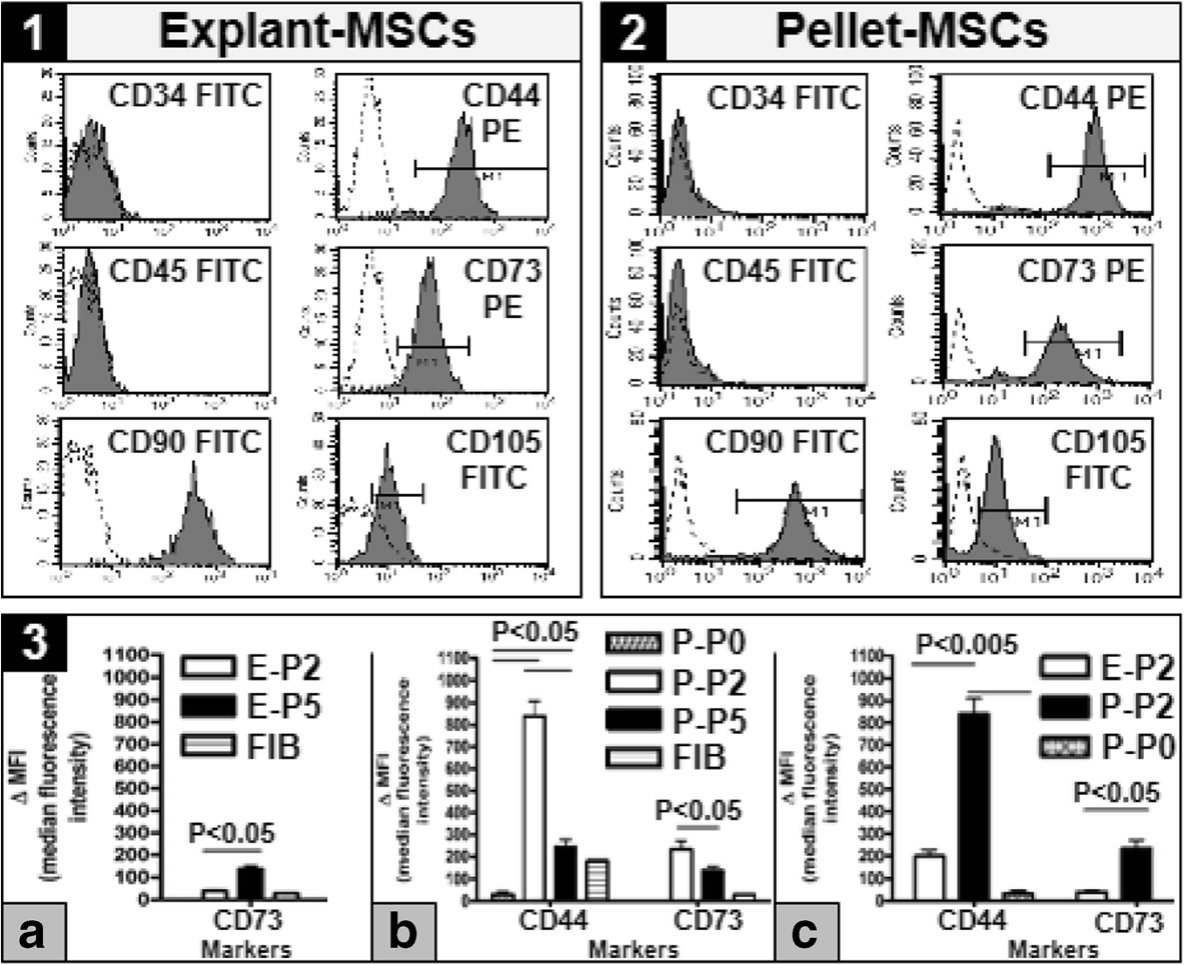
**Flow cytometry analysis of WJ explant- and pellet-derived MSCs cultured in XF medium.** MSC markers expression by WJ explant **(1)**- and pellet **(2)**-derived MSCs at P2; **(3)** Comparative MFI expression of CD44 and/or CD73 markers in: **(3a)** Explant-derived MSCs at P2 (E-P2) versus P5 (E-P5); **(3b)** Pellet-derived MSCs at P2 (P-P2) versus P5 (P-P5), as compared to freshly isolated pellet-derived WJ cells (P-P0) and a commercially available NUFF1 human fibroblast cell line (FIB); **(3c)** explant-derived MSCs at P2 (E-P2) versus pellet-derived MSCs at P2 (P-P2), as compared to freshly isolated pellet-derived WJ cells (P-P0). MFI, median fluorescence intensity; MSCs, mesenchymal stem cells; P, passage; WJ, Wharton’s jelly; XF, xeno-free.

**Figure 6:**
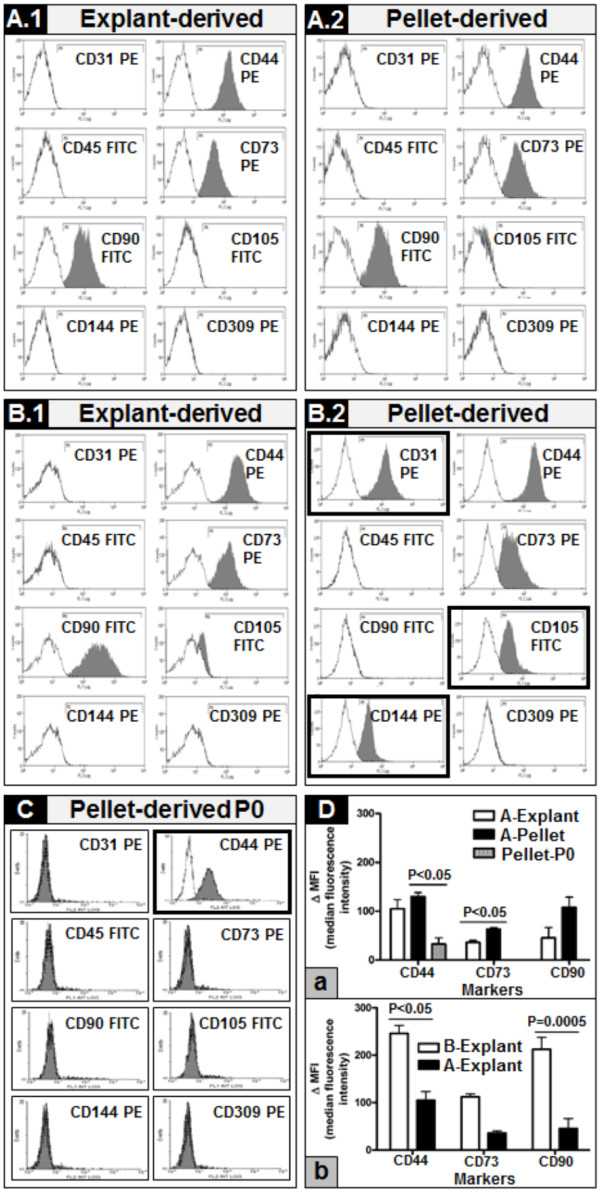
**Flow cytometry analysis of WJ-derived cells cultured in endothelial medium after isolation/expansion in different conditions. A.1** and **A.2 **- explant- and pellet-derived cells, respectively, belonging to group A, isolated and expanded until P2 in the XF medium, and then subcultured for five consecutive passages in endothelial medium, showed lack of endothelial surface markers; **B.1** and **B.2** - explant- and pellet-derived cells, respectively belonging to group B, isolated and subcultured for five consecutive passages in endothelial medium, showed differential expression of surface markers: explant-derived cells (B.1) did not gain endothelial surface markers expression, whereas pellet-derived cells (B.2) exhibited CD31, CD105 and CD144 surface markers expression upon culture into the endothelial medium; **C.** Surface markers expression on enzymatically dissociated, freshly isolated WJ cells (pellet-derived P0); **D.** Comparative MFI expression of CD44, CD73 and CD90 markers, upon exposure to endothelial differentiation conditions, in: **(D.a)** explant- versus pellet-derived cells belonging to group A (A), as compared to enzymatically dissociated, freshly isolated WJ cells (pellet-P0); **(D.b)** explant-derived cells from group B (B) versus explant-derived cells from group A (A). MFI, median fluorescence intensity; P, passage; WJ, Wharton’s jelly; XF, xeno-free.

### WJ-MSCs cultured in the XF medium did not subsequently differentiate into endothelial cells

**Figure 7:**
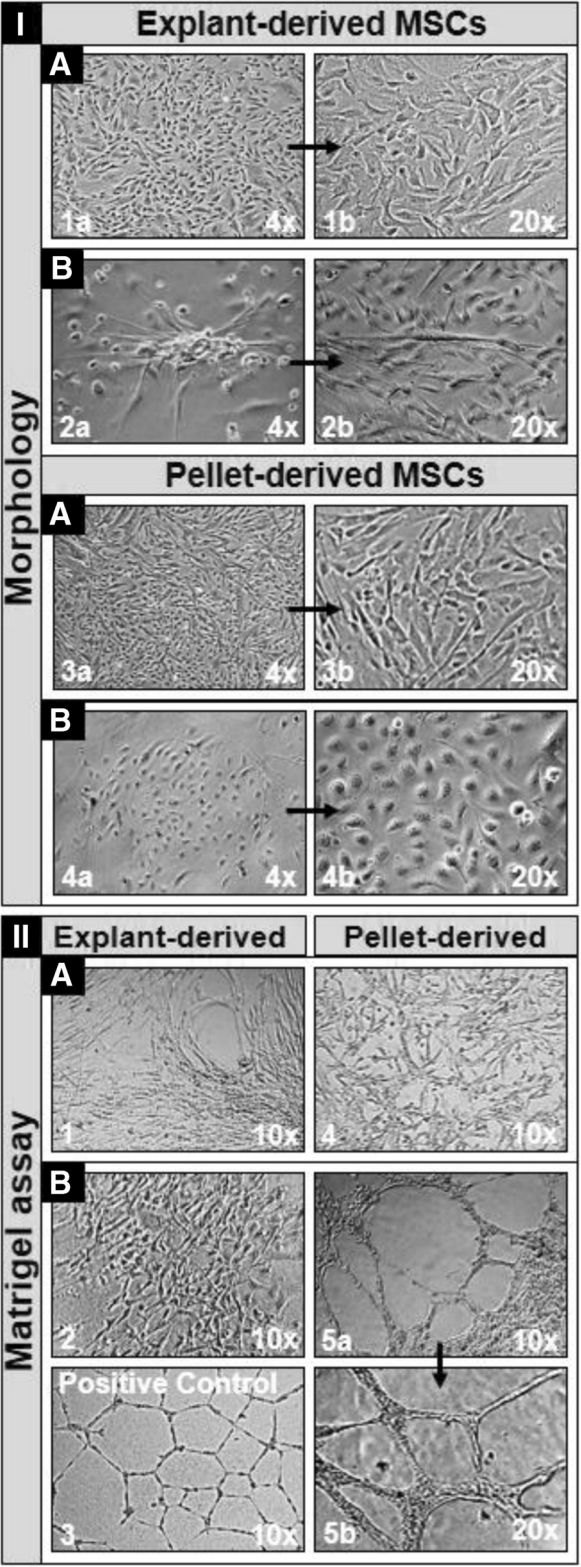
**Morphology (I) and tube formation capacity (II) of WJ-derived cells used into endothelial differentiation assays. A** – Group A cells isolated and expanded until P2 in the XF medium, and then subcultured for five consecutive passages in endothelial medium; **B** – Group B cells isolated and subcultured for five consecutive passages in endothelial medium. Explant- and pellet-derived cells belonging to group A did not show morphological (I.A – 1a/1b and 3a/3b, respectively) and functional (II.A – 1 and 4, respectively) features of endothelial cells. Within group B, explant-derived cells also neither exhibited morphological (I.B – 2a/2b), nor functional (II.B – 2) characteristics of endothelial cells, whereas pellet-derived cells presented endothelial characteristics both at morphological (I.B – 4a/4b) and functional (II.B – 5a/5b) levels; II.B – 3, positive control for Matrigel tube formation assay, represented by a commercially available HUVEC line. Higher magnifications of corresponding pictures are pointed by arrows. HUVEC, human umbilical vein endothelial cell; MSCs, mesenchymal stem cells; P, passage; WJ, Wharton’s jelly; XF, xeno-free.

In order to investigate if the WJ-MSCs isolated and expanded in the XF medium could differentiate into endothelial cells, we performed endothelial differentiation assays. Upon exposure for five serial passages to endothelial differentiation medium, both WJ explant- and cell pellet-derived MSCs isolated and expanded until P2 in the XF medium (group A), presented a fibroblast-like morphology (Figure 7.I.A.1a/1b and 3a/3b, respectively) and did not form tube-like structures upon culture on Matrigel basement membrane matrix (Figure 7.II.A.1 and 4, respectively). By contrast, adherent cells from group B, isolated and subcultured for five consecutive passages in endothelial differentiation conditions, derived from WJ cell pellets, developed an epithelial-like morphology and presented functional characteristics of endothelial cells by forming vascular tube-like structures in Matrigel (Figure 7.I.B.4a/4b and II.B.5a/5b, respectively), while the WJ explant-derived counterparts maintained a fibroblast-like morphology and did not exhibit tube-forming characteristics (Figure 7.I.B.2a/2b and II.B.2, respectively).

Furthermore, flow cytometry analyses indicated that both explant- and pellet-derived cells belonging to group A did not express endothelial surface molecules and maintained the expression of CD44, CD73 and CD90 MSC markers, while losing CD105 marker expression (Figure 6.A.1 and A.2, respectively); however, WJ pellet-derived cells showed a statistical significantly higher (*P* < 0.05) CD44 expression than freshly isolated WJ-derived cells (P0) and a statistical significantly higher (*P* < 0.05) CD73 expression, as compared to explant-derived cells (Figure 6.D.a). Within group B, explant-derived cells also did not show endothelial surface molecules, but expressed CD44, CD73 and CD90 MSC markers (Figure 6.B.1). Interestingly, the pellet-derived cells belonging to group B presented a high expression of CD44 and CD73, but not CD90 markers; furthermore, they presented an endothelial phenotype through expression of CD31, CD105 and CD144 surface molecules (Figure 6.B.2). When we compared MSC markers expression between groups A and B, the only statistically significant differences applied to explant-derived cells, whose CD44 and CD90 expression was higher (*P* < 0.05 and *P* = 0.0005, respectively) in group B, as compared to group A cells (Figure 6.D.b).

Moreover, quantitative gene expression of *vWF* and *CD31* endothelial markers did not significantly increase in group A cells, as compared to controls (Figure 4.2a and 2b, respectively). By contrast, pellet-derived cells belonging to group B had an increased quantitative mRNA expression for *vWF* and *CD31* markers (Figure 4.2c and 2d, respectively), as compared to enzymatically dissociated, freshly isolated WJ cells (*P* < 0.05) and cells from group A at a corresponding passage. The gene profile of cells belonging to groups A and B was further documented by RT-PCR, showing that only enzymatically dissociated, freshly isolated WJ cells (P0) and pellet-derived cells belonging to group B expressed *CD31*, *CD144* and *vWF* markers involved in endothelial cells differentiation, proliferation and survival, as well as *GATA2* transcription factor that promotes angiogenesis [40]; *VEGFR1*, with a role in stem cell recruitment [41], chemokine receptors *VEGFR2* and/or *Tie-2*, important in stem/progenitor cells homing were also shared by cells belonging to group A and Explant-derived cells belonging to group B (Figure 4.3).

Overall, the results of endothelial differentiation assays indicated that the endothelial differentiation capacity of WJ-derived adherent cells depended on the cell isolation method and initial culture conditions.

## Discussion

In this study, we report that the use of a selective, XF culture medium, specifically designed for MSCs, led to the isolation from human WJ explants and enzymatically dissociated WJ of cell populations with MSC characteristics as assessed at gene, protein and functional levels, which were differently expressed according to the cell isolation method used. Furthermore, we demonstrated that the initial culture conditions, used for the isolation and expansion of WJ-MSCs, resulted in lack of subsequent endothelial differentiation potential. Therefore, the use of defined MSC culture media is a prerequisite for the establishment of the real capacity of WJ-MSCs to differentiate towards the endothelial lineage.

The WJ-derived cells isolated and expanded in a MSC XF medium proved to present a robust MSC profile through expression of CD44/CD73/CD90/CD105 surface markers, multipotent differentiation potential into adipogenic, osteogenic and chondrogenic cell lineages, as well as a high proliferation rate. When BM-MSCs [26,42,43], which expressed less growth-related genes as compared to WJ-MSCs [44], and adipose stem cells [25] were manipulated *in vitro* by using XF media, they proliferated more rapidly than the cells cultured by using conventional media. In addition, MSCs derived from enzymatically-digested whole human umbilical cord segments [45] and umbilical cord matrix [46] had a higher expansion potential when cultured in serum-free/XF media, as compared to FBS-based media cultured counterparts and BM-MSCs/adipose stem cells, respectively. Although we did not compare in this study XF versus conventional culture media, our results corroborated with the literature data further indicate that the XF conditions described here might bring an advantage over the conventional media, in respect of proliferation properties of WJ-MSCs.

We also demonstrated significant differences in CD44, CD73 and CD90 surface marker expression in WJ-MSCs cultured in XF conditions, as well as in WJ-MSCs and WJ-derived adherent cells exposed to endothelial differentiation conditions, depending on the cell isolation method used or the passage number. It is well known that both *in vitro* cell manipulation technique [47] and passage number [48] exert a prominent impact on the protein expression profile of MSCs. The issue of heterogeneity in MSC markers expression has also been reported for BM-MSCs and has been correlated with their proliferation and differentiation potential [49]. It has been shown that CD44, CD73 and CD90 markers were expressed at higher levels in umbilical cord-derived MSC subpopulations that exhibited a higher proliferation capacity and a reduced amount of aging cells [50]. In addition, CD44, a cell-surface receptor for hyaluronic acid, facilitated MSCs migration through interaction with extracellular hyaluronic acid, suggesting that such migratory mechanism could be critical for MSCs recruitment to tissue regeneration sites [51]. As CD44 and CD90 are involved in cell-to-cell interactions, cell adhesion and migration, and CD73 plays an important role in cell cycle progression and apoptosis, they might differently control these processes in WJ explant- versus pellet-derived cells, nevertheless by passage propagation. Therefore, the biological benefits of such heterogeneity in the level of surface markers’ expression may be related to the proposed functions of these cells. Further insights into these markers’ expression by WJ-MSCs manipulated in XF culture conditions are worthwhile in order to modulate biological processes, such as cell adhesion, migration and proliferation, to optimize clinical cell therapy approaches. Thus, the development of protocols for isolation of distinct MSC subpopulations, based also on the expression levels of their surface markers, may provide improved vectors for the treatment of specific diseases [52].

When assessing multipotent differentiation outcomes, we demonstrated that in WJ explant- and pellet-derived MSCs, the chondrogenic and osteogenic differentiation potential increased and decreased, respectively, by passage progression. This could be due to the fact that the MSCs isolation method used (via WJ explants versus enzymatic dissociation of WJ) led to the generation of different MSC populations in respect of their multipotent differentiation capacity. It has also been documented that the passage number has an important impact on the differentiation capacity of MSCs [48,53,54] due to a consistent pattern of changes in the global gene expression signature of MSCs at different passages [48]. In this respect, MSCs derived from other sources than WJ, either exhibited a decreased osteogenic differentiation capacity by passage progression [54] or calcium deposition transiently decreased from P4 to P6, but returned to levels near or above those of primary cells by P10 [55]. Therefore, further work is needed in order to elucidate the optimum isolation protocol and passage number of WJ-MSCs, grown in defined, XF conditions, to optimize their multipotent differentiation potential according to the clinical cell therapy foreseen.

Although it has been shown that WJ-MSCs cultured in conventional media presented a lower adipogenic differentiation potential as compared to other MSC sources, such as the BM [44], periodontal ligament [56] and chorionic-plate [57], we found that WJ-MSCs isolated and expanded in the XF medium presented different degrees of adipogenic differentiation, according to the isolation method and passage number; interestingly, the adipogenic differentiation capacity of WJ pellet-derived MSCs was higher at P5 as compared to P2 and was inverse proportional with the osteogenic differentiation potential of these cells. This may be due to the involvement of MicroRNA-22 that was found to regulate adipogenic and osteogenic differentiation of human adipose tissue-derived MSCs in opposite directions [58], a finding that deserves to be further investigated in WJ-MSCs, too.

While it has been reported that both the osteogenic gene expression pattern [44] and osteogenic differentiation rate [59] were lower in WJ-MSCs as compared to BM-MSCs, isolated by using conventional media, we demonstrated a very robust osteogenic differentiation potential of WJ explant-derived MSCs, which increased by passage progression. Furthermore, the influence of the culture conditions used for MSCs isolation and expansion on gene and protein expression profile of BM-MSCs [47], as well as on their osteoblastic differentiation has been demonstrated. In this regard, XF conditions used for BM-MSCs culture, through FBS substitution for allogeneic human platelet lysate, enhanced their osteogenic differentiation [43]. Hence, the WJ-MSCs isolated and expanded by using the selective, XF conditions described here might be better cell candidates for bone regeneration, as compared to WJ-MSCs manipulated in conventional media, suggesting potential superior *in vivo *osteogenic regeneration outcomes. In respect of the impact of the XF conditions on chondrogenic differentiation potential, it has been shown that BM-MSCs that were isolated, stored and expanded using XF materials, including the XF medium used in our study, had a higher gene expression of *aggrecan* than cells cultured in conventional media [42]. These observations indicate that WJ-MSCs, manipulated *in vitro* by using XF media, might also represent superior cell candidates for cartilage repair.

Upon exposure to endothelial differentiation signals, we showed that both WJ explant- and pellet-derived cells from Group A - isolated and expanded until P2 in MSC XF medium, and then subcultured for five passages in endothelial differentiation conditions - did not exhibit endothelial differentiation potential. By contrast, pellet- but not explant-derived cells belonging to Group B - isolated and expanded in endothelial differentiation conditions from P0 to P5 - presented endothelial cell characteristics, being different in respect to surface and gene marker expression, as well as functional properties. This might be due to the fact that the enzymatically dissociated, freshly isolated WJ cell populations contained stem/progenitor cells or other contaminating endothelial cell types able to give rise to an endothelial progeny, when seeded directly into endothelium medium for five passages; in the case of explant-derived cells of group B, these cells may not migrate out of the explants. Furthermore, they may be lost when initially cultured in XF conditions.

Interestingly, we also demonstrated that, besides the endothelial markers expression at gene and protein levels, the WJ pellet-derived cells belonging to group B presented CD73 and CD44 MSC surface markers. Although there are no data yet documenting CD44 marker expression on endothelial cell outgrowth derived from WJ, it has been reported the involvement of CD44 molecule in endothelial cell proliferation, migration and angiogenesis [60], contributing to the organization and/or stability of developing endothelial tubular networks [61]. While there is also no evidence of CD73 expression on endothelial progenitors derived from any adult tissues, Choi KD *et al*. [62] identified a novel population of CD73+ endothelial progenitors derived from human embryonic stem cells. These data and the fact that the umbilical cord is closer in development to embryonic than adult tissues [44,57], reinforce the speculation that the isolated endothelial progeny within pellet-derived cells belonging to group B resulted from a different stem/progenitor cell type than MSCs, residing in the matrix of the umbilical cord, whose isolation might have been facilitated by the use of enzymatic dissociation and initial endothelial culture conditions.

Although some reports have demonstrated endothelial differentiation of WJ-derived cells *in vitro* or *in vivo*[13,16-19], endothelial differentiation of WJ-MSCs is still very controversial. Furthermore, it has been shown that MSCs derived from other tissues than WJ, such as BM and adipose tissue, did not display functional properties of endothelial cells, but promoted neovascularization via paracrine mechanisms [63,64]. Choi M *et al.*[65] also demonstrated the lack of endothelial differentiation capacity of WJ-MSCs, isolated and expanded in a conventional FBS-based medium, but paying careful attention to the removal of the umbilical cord blood vessels and amnion. Upon exposure to endothelial differentiation media, these WJ-MSCs neither expressed endothelial markers, nor directly participated to angiogenesis/vasculogenesis *in vitro* and *in vivo*; rather, the cells improved perfusion recovery and neovascularization by secreting paracrine factors and by functioning as perivascular precursor cells [65]. Our data, corroborated to this report, suggest that the MSCs’ isolation method has a tremendous impact on the homogeneity of the cells and their true endothelial differentiation capacity. By contrast with other authors that did not use a selective MSC medium for WJ-MSCs isolation prior to their exposure to endothelial differentiation conditions [65] or cultured freshly isolated cells directly in endothelial differentiation media [17], we further documented, by using a defined, XF medium for MSCs, that the initial culture conditions have a strong impact on the progeny outgrowth resulted upon exposure of WJ-MSCs to endothelial differentiation signals. Based on our observations, we speculate that in the case of previous reports, describing WJ-MSCs differentiation into endothelial phenotypes, the endothelial outgrowth was derived from other types of stem cells than WJ-MSCs, circulating endothelial progenitors, or umbilical vein endothelial cells, contaminating the primary cultures during the dissection process, performed to remove the umbilical cord arteries and the vein.

It is well known that gene expression profiling is significantly different among MSCs from distinct sources and that intrinsic gene expression has an important impact on MSCs overall differentiation potential [66]. It has been postulated that BM-MSCs constitutively expressed genes related to immunomodulation, adipose tissue derived-MSCs highly expressed genes implicated in tissue development [14], whereas the transcriptome profiling of WJ-MSCs revealed an increased expression of genes involved in liver development [66] and an inherent bias towards the neuro-ectoderm lineage [14,67]. These observations suggest that WJ-MSCs might be better cell candidates for differentiation into hepatocyte and neuronal rather than endothelial phenotypes. As MSCs from different sources exhibit distinct and unique gene expression signatures, which make them competent to give rise to specific lineages rather than others [14], at this moment there are no well-defined master MSCs that are suitable for vascular regeneration. Therefore, according to the isolation and culture methods used, MSCs should be rigorously characterized, especially at the gene and functional levels, and much caution needs to be given when choosing the best MSC type for a particular therapeutic application.

## Conclusions

Taken together, our data indicate that the isolation method and XF culture conditions influence the multipotent differentiation capacity of human WJ-MSCs. The use of a selective, XF medium for MSCs led to the isolation and expansion of MSC populations from the matrix of the umbilical cord, free of contamination with other stem, progenitor or adult cell types that may be misleading concerning the real endothelial differentiation potential of WJ-MSCs. Therefore, the availability of optimized *in vitro* assays, involving ready-to-use XF media for human WJ-MSCs manipulation, might have a substantial scientific and clinical potential. Thus, a priority in the field should be the standardization of isolation and culture expansion techniques in defined, XF media for an accurate characterization of WJ-MSCs, as candidates for clinical cell therapy applications.

## Abbreviations

BM: Bone marrow; BM-MSCs: Bone marrow-derived mesenchymal stem cells; FBS: Fetal bovine serum; FITC: Fluorescein isothiocyanate; GAPDH: Glyceraldehyde 3-phosphate dehydrogenase; HUVECs: Human umbilical vein endothelial cells; MFI: Median fluorescence intensity; MSCs: Mesenchymal stem cells; MTT: 3-(4,5-dimethylthiazol-2-yl)-2,5-diphenyltetrazolium bromide; P: Passage; PBS: Phosphate-buffered saline; PCR: Polymerase chain reaction; PE: Phycoerythrin; qRT-PCR: Real-time quantitative reverse transcription polymerase chain reaction; RT: Reverse transcription; RT-PCR: Reverse transcription polymerase chain reaction; VEGF: Vascular endothelial growth factor; VEGFR: Vascular endothelial growth factor receptor; vWF: von Willebrand factor; WJ: Wharton’s jelly; WJ-MSCs: Wharton’s jelly-derived mesenchymal stem cells; XF: Xeno-free.

## Competing interests

The authors declare that they have no competing interests.

## Authors’ contributions

MCC participated in cell culture experiments, differentiation, histochemistry, molecular biology and Matrigel assays, and performed statistical data analysis. MAP participated in cell culture experiments, differentiation, molecular biology and Matrigel assays, and performed flow cytometry sample preparation and statistical data analysis. AR participated in cell culture experiments, differentiation, histochemistry and MTT assays, and performed statistical data analysis. LES performed flow cytometry data acquisition and statistical data analysis. GI collected the umbilical cords,performed virology testing, and critically revised the manuscript. MLP participated in experiments, designed and coordinated the study, interpreted the data, wrote the manuscript, and gave final approval for manuscript publication. All authors read and approved the final manuscript.

## Authors’ information

MLP, Ph.D. is the leader of the Angiogenesis and Cardiovascular Remodeling Group at the Institute of Cellular Biology and Pathology “Nicolae Simionescu” in Bucharest and is a founding member of the Romanian Society for Developmental Biology. MLP coordinates national and international projects,including FP7, in the field of adult stem and progenitor cells, with a focus on cellular and molecular mechanisms of vasculogenesis and cardiovascular regeneration.
